# COVID-19 Pandemic & Bureaucracy: The Crisis Inside the Crisis

**DOI:** 10.3389/fpubh.2021.665323

**Published:** 2021-10-07

**Authors:** Katalyn Roßmann, Heike Wegner, Hans Stark, Gerd Großmann, Andreas Jansen, Dimitrios Frangoulidis

**Affiliations:** ^1^Bundeswehr Medical Service Headquarters VI-2, Medical Intelligence & Information (MI2), Munich, Germany; ^2^Bundeswehr Centre for Software Expertise (BwCSE), Euskirchen, Germany; ^3^Federal Information Centre for International Health Protection, Robert Koch-Institute, Berlin, Germany

**Keywords:** COVID-19, Cynefin framework, crisis, innovation, layperson training, evaluation matrix, public health dashboard, Bundeswehr

## Abstract

The Medical Intelligence and Information (MI2) Unit of the German Armed Forces (Bundeswehr) is experienced in crisis support in military missions since several years. It gained additional experiences during the current coronavirus 2019 (COVID-19) pandemic on different levels of the response to crisis and was requested to share the findings and expertise with the overloaded civil public health agencies inside Germany. Since the beginning of the pandemic, the unit is constantly developing new products for crisis communication, knowledge sharing techniques in new databases, dashboards for leadership, and training for laypersons in contact tracing. Hence, trying to innovate in crisis since the first severe acute respiratory syndrome coronavirus (SARS-CoV)-2-disease wave. During the second wave, the unit was requested to evaluate the outbreak management of different national civil public health agencies in southern Germany, and to support the development of dashboards in a comprehensive public health approach as a necessary start toward digitalization.

## Introduction

As seen in former times, management of infectious diseases is very often a challenge and especially when more people and regions are involved, and containment takes time. This is standard knowledge of infectious disease and public health specialists. This information became more aware to the public and to the decisionmakers worldwide during the Ebola-crisis in West-Africa 2014—when countries were locked down due to medical, social, economic, and governmental/political reasons. As one of the consequences, the Global Health Security (GHS) index was developed to score the ability of countries to manage a pandemic infectious crisis (1). The first test of the validity of this index is now realized during the coronavirus disease 2019 (COVID-19) crisis, the first pandemic in the past 100 years.

The core of managing a country is the organizational workflow in administrations, summarized as bureaucracy.

Bureaucracy can be defined as the most efficient of all known organizational systems. However, on the downside, it lacks power for innovation and does not stimulate systemic learning aptitude or interdepartmental coordination (2). Realizing the overwhelming and broad challenges of the ongoing COVID-19-pandemic, we must ask: Is our bureaucratic health administration adequately organized to manage a pandemic crisis? Or do we have to change our strategy?

**Figure 1:**
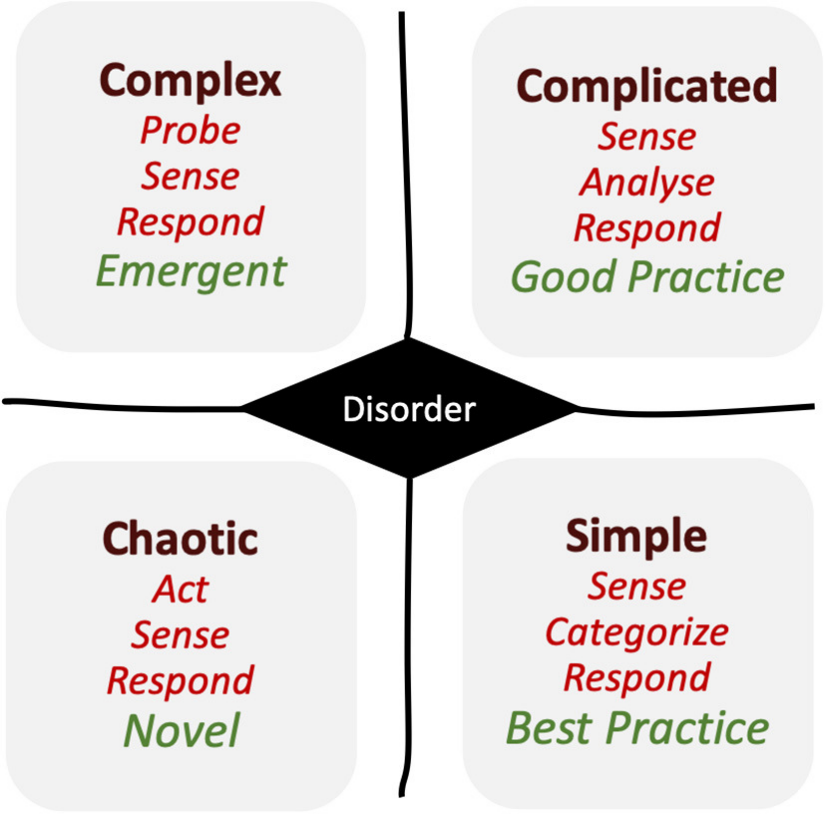
Cynefin framework [modified from Snowden, (3, 4)].

To answer this question, the Cynefin framework (3–5) (Figure 1) proves to be helpful. This conceptual framework is used to aid decision-making and offers a possible categorization, which can be reasonably applied to situations similar as the current corona crisis.

Briefly (as shown in Figure 1): If the situation or status is “simple”—in the sense of solid, steady, or stable—then the standard “best practice” is achievable and bureaucracy functions without any difficulties at its best. If the situation or status changes to “complicated”—which is defined by solvable problems—then the bureaucracy still works, because the challenges can be overcome. In this case, the standards might be downsized from “best to good.” Nevertheless, this seems to be acceptable.

However, if the situation changes into “complex,” and is defined by the problems which cannot be solved easily anymore, the reaction has to be somehow “emergent.”

Finally, if the situation becomes “chaotic,” we do not know the number of problems we have in a given place—and whatever we need to do seems to be novel and innovative.

As long as the problems are solvable, the bureaucracy and its administration operate well. However, as the situation deteriorates, experienced organizations in the crisis management are requested to intervene. Hence, that is the reason why firefighters and military forces are often activated during the crises and catastrophes. The latter must fill the quantitative gaps for the necessary additional workload in crisis management (e.g., contact tracing or logistics), but also qualitatively as the situation room operating forces with intuitive leadership competence, georeferenced, and statistical dashboards as digital knowledge management capacities. According to the above-mentioned framework, the innovative and agile forces are needed to manage crises—such as the current COVID-19 pandemic. Below, we have presented and identified four areas of concern, improvements, and tools for crisis support:

1. Information-management including crisis communication

2. Data- and information-visualization (dashboard)

3. Training and education of supporting staff

4. Framework and evaluation concept (“scoring-matrix”).

### Information-Management Including Crisis Communication

Gaining and circulating information in vague and complex settings—poor bureaucracy leading to an opportunity for innovation in crisis.

Since the beginning of the corona pandemic, the Public Health Intelligence Unit of the Robert Koch-Institute and the Medical Intelligence & Information (MI2) Unit of the German Armed Forces are reporting about this event in a weekly newsletter for German government agencies. Due to a new globally spreading virus and the lack of knowledge about it, another scientific Newsletter “Infekt-Info” was developed for medical experts in the German Armed Forces, including a “Journal Club” database for screening and reviewing the exponentially growing number of daily-published scientific papers worldwide (see www.gr-solutions.de). In a call for swarm, the intelligence medical experts were requested to support the daily workload of reviewing these articles. The latter newsletter was quickly requested by many organizations, public authorities, and medical personnel in other German-speaking countries. This informative service was offered during the first pandemic wave on a daily basis and is still running two times a week, by offering all the affiliated stakeholders in the medical and administrative field the relevant information and news about COVID-19 in Germany, Europe, and worldwide.

### Data- and Information-Visualization (Dashboard)

Coordination with information and data visualization (“MI2-Dashboard”).

As the number of new cases in Germany increased in spring 2020, the German Armed Forces with its MI2 Unit started to develop its first own dashboard derived and inspired from the worldwide used example of Johns Hopkins University to offer the military leaders an overview about the pandemic situation worldwide, in Germany and in the German Armed Forces.

Since the COVID-19 pandemic, dashboards have become the new trend, used worldwide and an accepted presentation standard for epidemiological associated data especially when they are Geoinformation System (GIS)-supported (6). Additionally, in the MI2 Unit, the need for such an online, updated visualization system was very early identified in the COVID-19 pandemic crises. Using Bundeswehr-based expertise from the Bundeswehr Center for Software Expertise (BwCSE), a Bundeswehr based system for the Medical Command was planned and shared in February 2020. The tool is designed to establish a convenient way to provide all military personnel with an accurate yet easy-to-grasp medical information for military decisionmakers in a secured area. The new information dashboard is hosted at the Bundeswehr Geoinformation Center (BwGIC) using an already existing server site and a licensed Portal for ArcGIS®–a professional commercial GIS-Software from the ESRI-Company. Named users can create their own applications, upload, and publish data using a variety of predefined tools, which allow a certain configuration at the same time.

**Figure 2:**
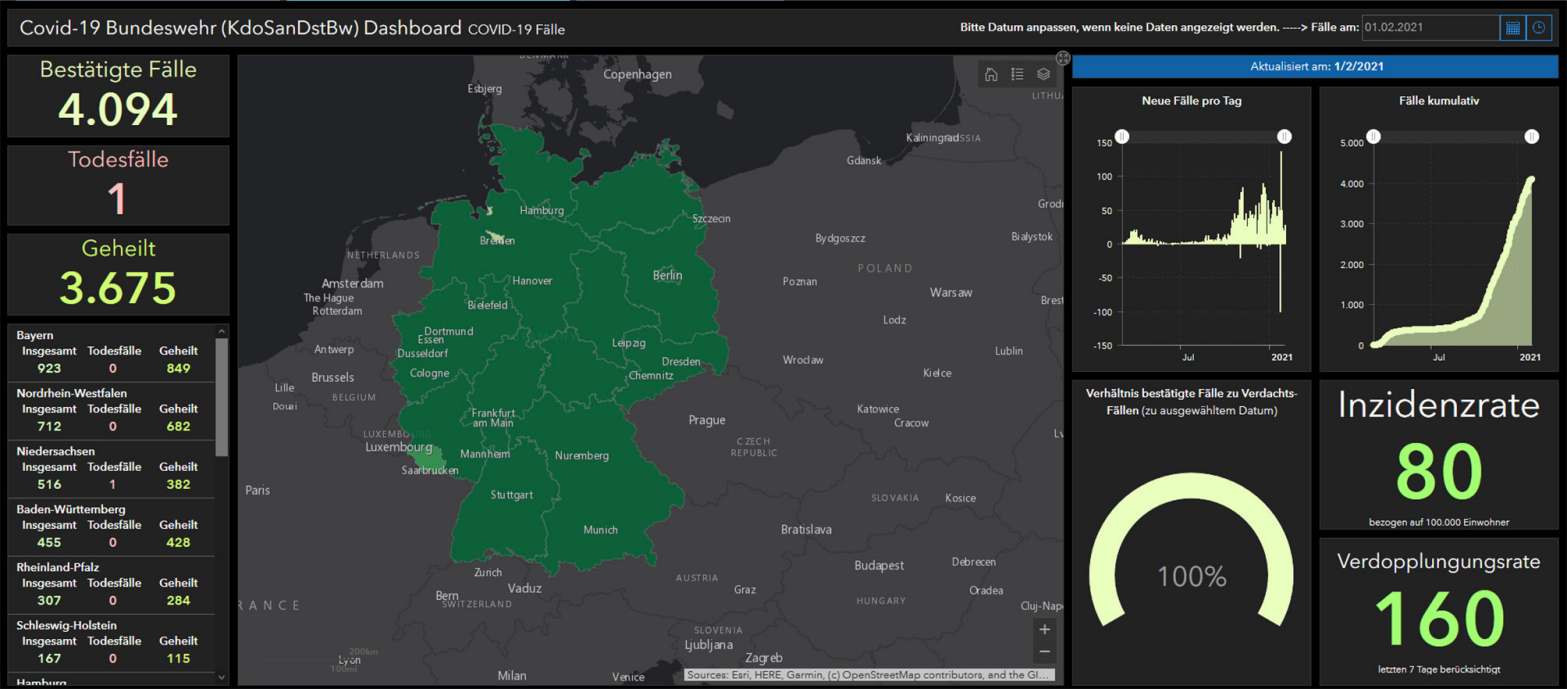
MI2 dashboard, Bundeswehr.

Information from different sources were aggregated and presented in a single graphically pleasing application, summarized in charts and bars while providing basic analytical features for logged-in Bundeswehr users with specialized medical backgrounds (as shown in Figure 2). Along the surveillance data from the Medical Command, additional data sources were automatically extracted from feature layers of the US Johns Hopkins University and from the German Robert Koch-Institute feature layers and several other data sources with information regarding the worldwide Corona infections.

While creating the Medical Command dashboard of the Bundeswehr, the important issues for the tool were identified. Presentation design is the final step of a successful dashboard, but to update all the data posed a real challenge—especially if different sources, data-standards, and formats are used. This also includes, simply, which type of browser is used. To reduce the number of persons involved, the primary aim was to develop automatic extraction and importation tools and an algorithm for these dashboards that could routinely be checked for updates.

The next step in information and data retrieval will be the introduction of artificial intelligence systems for data and text mining, as a recent published paper demonstrated (7).

All these dashboards are still under construction and the civil-military cooperation will continue developing them during the coming months of this pandemic. We are convinced that dashboards, also based on our suggested evaluation matrix (see below and Supplementary Material), are an important step in re-tooling workflows in public health—thus, supporting the transformation into a process-guided and evidence-based structure—a real incredible innovation in bureaucracy!

### Training and Education of Supporting Staff

Training laypersons due to lack of professionals in unclear and complex settings—another innovation in crisis according to routine processes in bureaucracy.

In spring 2020, the MI2 Unit additionally started to apply the WHO Primary Health Care Approach (8, 9) by training military musicians as community healthcare workers. Thus, training these laypersons for contact tracing (in Germany called: Containment Scouts) to support the overwhelmed civil public health agencies especially in contact tracing of COVID-19 cases. The Primary Health Care Approach is a worldwide known strategy, and the workforce of community health workers is well-established for decades. However, it is not a part of Germany's “luxurious” health care system. Shifting the standards from having all the time health professionals to trained laypersons was and is a drastic shift, and this is a possible and a necessary innovation in such a crisis. Meanwhile, it has become an accepted phenomenon during this crisis, e.g., in contact tracing, to support the medical care in overloaded clinics and elderly's and nursing homes which is conducted by military, police or firefighter forces, and other civil servants. The experts of the MI2 Unit have trained hundreds of soldiers as Containment Scouts while traveling across Germany and developed e-learning tools for many others. Many thousands have worked and are still working in those civil public health agencies throughout the country.

### Framework and Evaluation Concept (Scoring-Matrix)

The WHO frameworks to optimize Corona Crisis Management in Germany—stay creative and digitalize: The real challenge in bureaucracy.

In September 2020, and with the onset of the second wave of COVID-19, the MI2 unit of the Bundeswehr was requested to support and evaluate the outbreak management of the largest local Public Health Office of Germany in Munich, responsible for more than 1.5 million people.

Therefore, a new framework was developed (as shown in Supplementary Material), which was derived from the experiences of unit from the Ebola outbreak in West Africa as well as the WHO 2019 Novel Coronavirus (2019-nCoV) (10, 11):

The following topics for evaluation were identified as a cross-sectional approach (first column or in rows—as shown in Supplementary Material):

- Command/Control/Communication (C3) (Central direction knowledge management; Evaluation Outbreak Management)
- Epidemiology (Investigation, Surveillance, Prediction)
- Patient Care (Outpatient, at home/on call service; preclinically and in-patient care, nursing at home/in-patient)
- Laboratory/Testing (generally, risk-oriented testing, smart testing)
- Risk Communication (in general, organized civil society, trained medical personnel, Press/Media/Citizen Hotlines)
- Outcome/administrative execution
- Studies (research and development according to SARS-CoV-2/COVID-19),
- Health Promotion (e.g., nutrition, exercise, stress management, and addiction prevention)
- Vaccination

For each topic, the following criteria (columns) must be evaluated and achieved by using a traffic light system (red: nothing existing; orange: something existing, but not functional; yellow: functional under limitations; and green: functional):

1. Is there process optimization potential?
2. Quantity and quality as well as training/qualification of personnel?
3. Suitability and availability of material and IT?
4. Suitability and availability of infrastructure?
5. Concepts and regulations existing?
6. Cooperation partners?

Evaluating and technically supporting the local public health agencies in seven different settings in the German Federal States of Bavaria and Thuringia over many months underlined the necessity to lead and manage crises in a comprehensive and cooperative public health approach. The evaluation revealed, in all settings, the potential in processing optimization of contact tracing and in digitalization of public health agencies in general. Many agencies were still trying to document and manage contact and case tracing by using Word®-files and Excel®-sheets. Moreover, according to the Federal State principle of Germany, many of the nearly 420 local public health agencies in the country worked on individual and different IT solutions and standards.

Therefore, together with one urban and several rural agencies, we developed indicators for a comprehensive and cooperative “Corona-Public Health-Dashboard” to get a better overview for local decision- and policymakers and to better manage the crisis and coordinate countermeasures. Previously, the available dashboards focused on the epidemiological aspects, as they were normally easier to present in common georeferenced systems. However, from a public health perspective as well as the perspective of a local policymaker, many more aspects should be available in one intuitive system. Therefore, same categories of our matrix-table (Command/Control/Communication (C3), Epidemiology (Investigation, Surveillance, Prediction), Patient Care, Laboratory/Testing, Risk Communication, Outcome/administrative execution, Studies, Health Promotion, Vaccination) as listed in the “Evaluation Framework” were brought together and became the dashboard files. For each topic and file, the stakeholders were identified, and indicators were discussed in the different agencies during virtual sessions or workshops onsite. This centralized, scalable, dynamic, and flexible central information dashboard is probably the ultimate tool in crisis management to get an overview of the situation.

## Discussion and Conclusions

To manage all aspects of a pandemic crisis, such as COVID-19 is a huge burden and challenge for countries, their government, administration, and policy and decisionmakers (12). Since then, a lot of suggestions, new insights, and ideas were shown, developed, and published to enhance the crisis management. Our experience in several public health agencies, from the smallest to the biggest, is that the problems to manage the COVID-19-crisis are very similar in the different places. There is no established bureaucratic workflow on how to perform in such a situation. Of course, there are plans on how to react to other crises or emergencies, such as floods or bus accidents. However, the immense challenge of a pandemic to coordinate and structure all parts of a society for a longer time, are not included. We realized that our tools were a very successful support and contributed to the improvement in managing the crisis. Meanwhile, several publications confirmed our tools. The use of mobile apps for a better information flow and management (13), is shown in our “Information-Management” part and also important for data- and information-visualization. The evaluation of government performance using mediation of government actions (14) and the enhancement of response plans with federated learning to accumulate the insights from multiple data source efficiently (7) is included in our framework and evaluation concept (scoring-matrix). Lu et al. (15) demonstrated the importance of having a well-organized planning and implementation of typical anti-epidemical countermeasures, such as lock-downs, large-scale suspension of business and schools, strict stay-at-home orders, widespread testing, and social distancing, we demanded support from decisionmakers. The general importance of ethical leadership e.g., “Information-Management including Crisis Communication,” was described here too (16). Another aspect is the weight of the influence of additional factors, such as quality of healthcare sector and environmental sustainability (17) that is also part of our scoring matrix.

On the other hand, we also acknowledged that the concept of prevention, an important factor of public health, is poorly considered in the governmental and administrative process. Therefore, the most important aim must be to gain sustainability for the future, being better prepared not only for the possible next wave of COVID-19, but also for the next pandemic crisis.

A more global perspective is seen in the former mentioned GHS index. Before the COVID-19 crisis, it was expected that a high-level rank of the GHS index, seen in the industrialized wealthy countries with a well working bureaucracy, will be a provision against the effects of a health crisis. Unavoidably, this was not the case due to the COVID-19 burden as shown by Aitken et al. (18). Therefore, some improvements were suggested, such as introducing new indices to quantify the environmental risk of exposure (19) or adapting the GHS index (20). But referring to indices shows pitfalls as Kaiser et al. demonstrated recently (21). They ended up in the recommendation “Keep it simple” as a hypothesis that we may not need more sophistication in the construction of global composite indicators, than just acting preventatively and quickly.

This brings us back to the message of our paper concerning the crisis of bureaucracy in the crisis and the not too sophisticated tools we described to overcome the problems.

By learning from past and present, we are offered the chance to implement tools and concepts to adapt, change and innovate the management system as discussed above. Here are the presented main areas for change and innovation (information-management/crisis communication, data- and information-visualization (“Dashboard”), training and education of supporting staff, and framework and evaluation concept (“Scoring-Matrix”) will offer policy and decisionmakers a fast and a suitable way to react to and in crisis situations. Therefore, we conclude:

### Changing the Perspective of the World

Now from north to south—applying international strategies, such as the WHO Primary Health Care Approach, which was developed for crisis shaken regions. Africa has decades of experience in managing health crisis situations and a well-established workforce of Community Health Workers alongside the limited—compared to many richer regions of the world—health professionals. In spring 2020, the idea to train laypersons for contact tracing in Germany sounded odd, but from a public health perspective and based on years of experience in developing countries, it was a necessary approach—as experts were already expecting the scary next wave.

### Bureaucracy and Its Limitations in Crisis

We experienced incredibly busy, highly stressed personnel in the civil administration. All of them were exhausted after almost a year of extraordinary situations and some crisis management with improvised structures in the traditional bureaucracy environment. Innovation, agility, flexibility, teamwork, and the art of improvisation in crisis persuaded most. The most important cooperation we experienced were those of public health experts with crisis management experts and IT-specialists. In each setting of those civil public health agencies, we convinced policy and decisionmakers—or at least tried to—create such teams to start more coordinated cooperation and digitalization. This included information and knowledge management, process and organizational optimization, and the implementation of new software, e.g., for contact tracing and georeferenced dashboards.

We ask in smaller as well as in larger settings during the actual second wave: Do we need to change the strategy? From our perspective, we are convinced: Well, applying cooperative and comprehensive public health would be enough. Nevertheless, to constantly innovate in crises—digitalize.

## Data Availability Statement

The original contributions presented in the study are included in the article/Supplementary Materials, further inquiries can be directed to the corresponding authors.

## Author Contributions

KR, AJ, HW, HS, and DF wrote the manuscript. HW and HS were responsible for the dashboard development and description. GG created and supported the Journal Club database. All authors did a critical review of the manuscript.

## Conflict of Interest

The authors declare that the research was conducted in the absence of any commercial or financial relationships that could be construed as a potential conflict of interest.

## Publisher's Note

All claims expressed in this article are solely those of the authors and do not necessarily represent those of their affiliated organizations, or those of the publisher, the editors and the reviewers. Any product that may be evaluated in this article, or claim that may be made by its manufacturer, is not guaranteed or endorsed by the publisher.
