# Supplementary material for: COVID-19 Pandemic & Bureaucracy: The Crisis Inside the Crisis
Source: Front Public Health. 2021 Oct 7;9:665323. doi: 10.3389/fpubh.2021.665323 (PMC8528952; doi:10.3389/fpubh.2021.665323)
Supplement: Supplementary file 2 [file Presentation_1.pptx]

## Slide 1
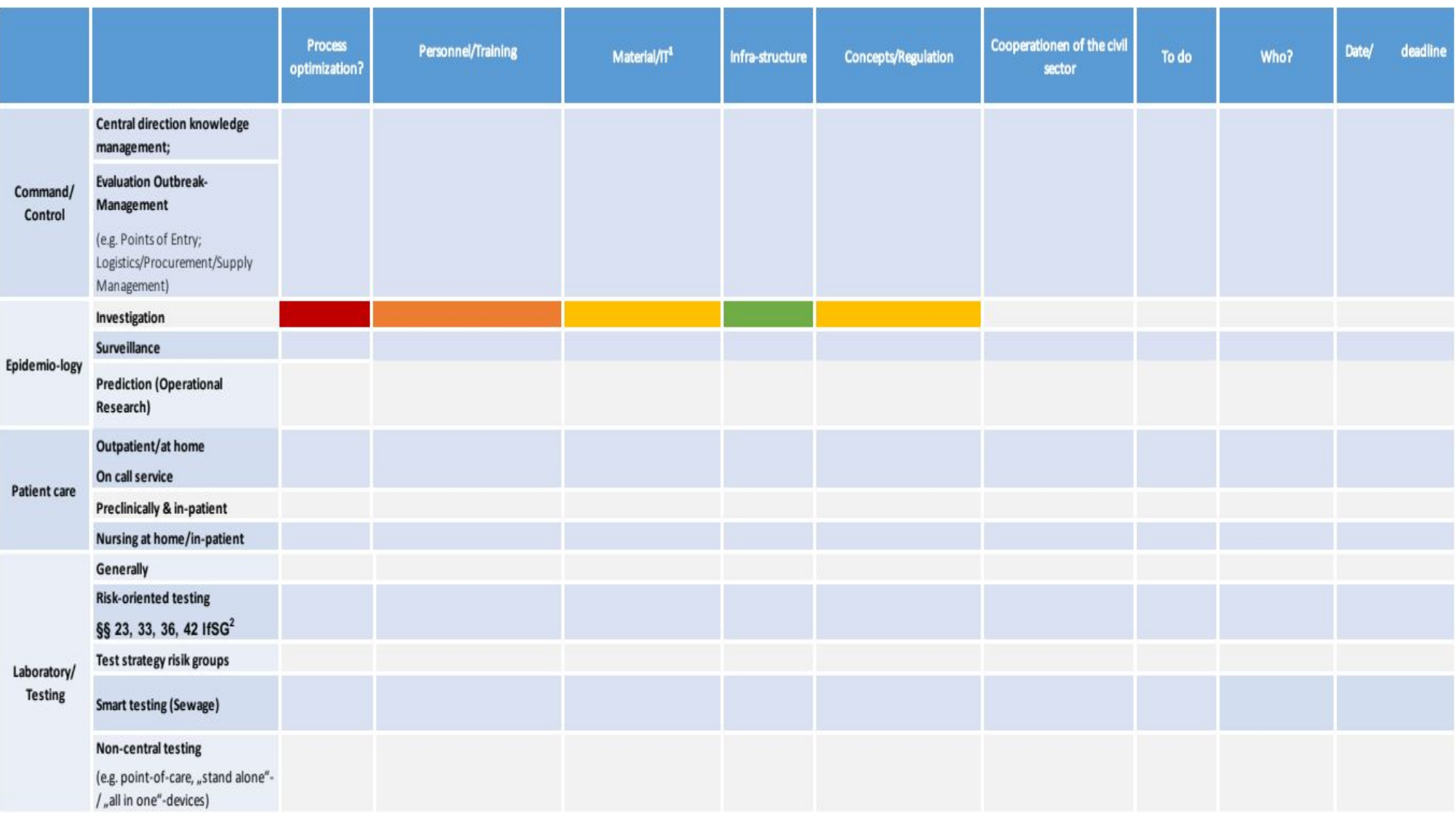

## Slide 2
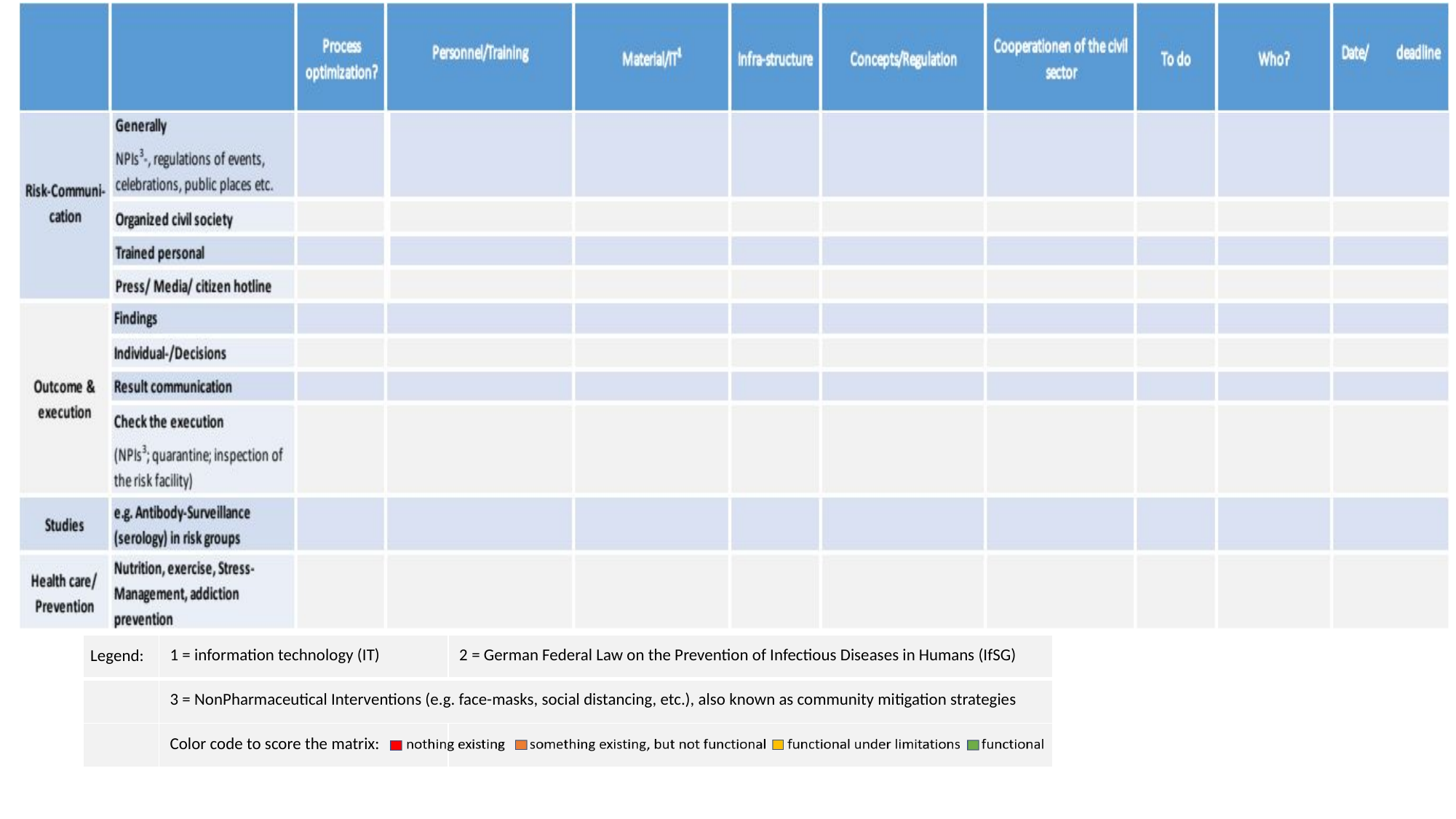

| Legend: | 1 = information technology (IT) | 2 = German Federal Law on the Prevention of Infectious Diseases in Humans (IfSG) |
| --- | --- | --- |
| | 3 = NonPharmaceutical Interventions (e.g. face-masks, social distancing, etc.), also known as community mitigation strategies | |
| | Color code to score the matrix: | |
